# The association between the workload of emergency physicians and the outcomes of acute myocardial infarction: a population-based study

**DOI:** 10.1038/s41598-023-48150-0

**Published:** 2023-12-01

**Authors:** Chang-Hung Tsai, Pei-Tseng Kung, Shun-Mu Wang, Tung-Han Tsai, Wen-Chen Tsai

**Affiliations:** 1 Miao-Li General Hospital, Ministry of Health and Welfare, Miaoli City, Taiwan, ROC; 2 Department of Health Services Administration, China Medical University, No. 100, Section 1, Jingmao Road, Beitun District, Taichung, 406040 Taiwan, ROC; 3 Department of Public Health, China Medical University, Taichung, Taiwan, ROC; 4 Department of Senior Services Industry Management, Minghsin University of Science and Technology, Hsinchu, Taiwan, ROC; 5 Department of Healthcare Administration, Asia University, Taichung, Taiwan, ROC; 6 Department of Medical Research, China Medical University Hospital, China Medical University, Taichung, Taiwan, ROC

**Keywords:** Health policy, Health services, Epidemiology, Cardiovascular diseases

## Abstract

Acute myocardial infarction (AMI) is the second leading cause of mortality in Taiwan. The correlation between the workload of emergency physicians and the outcome of AMI remains unknown. To determine the effects of the workload of emergency physicians on the outcomes of AMI. We included 17 661 patients (age > 18 years) with STEMI undergoing PCI, who visited the emergency department between 2012 and 2018. We used the logistic regression model with generalized estimating equations (GEEs) to analyze the risk of death within 30 days after emergency department visit, the risk of emergency department revisits within 3 days, and the risk of readmission within 14 days in all subgroups. After covariate adjustment, the risk of mortality within 30 days after visiting the emergency department was significantly higher in the subgroup whose visiting emergency physicians had the highest workload (odds ratio [OR]: 1.39; 95% confidence interval [CI]: 1.12 to 1.72). Furthermore, the risk of revisiting the emergency department within 3 days after discharge from the hospital was significantly higher in the subgroup whose visiting emergency physicians’ workload was within the second and third quartiles (OR 1.85; 95% CI 1.18 to 2.89). The workload of emergency physicians appears to be positively correlated with the mortality risk of patients with STEMI undergoing PCI.

## Introduction

Acute myocardial infarction (AMI) is the second leading cause of death in Taiwan. In 2021, the death toll due to AMI reached 184 172, and the an average death toll was 784.8 per 100 000 people^1^. In the United States, approximately 550 000 patients are newly diagnosed with myocardial infarction (MI) each year, and approximately 200 000 patients experience the recurrence of MI^2^.

Several studies have explored the correlations of the annual volume of percutaneous coronary intervention (PCI) performed by hospital offering PCI services^3–6^, and the annual and cumulative volume PCI performed by surgeons with the rate of mortality in patients with AMI^3–5,7,8^. The cumulative volume of PCI indicates the experience of PCI surgeons, whereas the annual volume of PCI indicates the sustained proficiency of PCI surgeons. For both the aforementioned parameters, certain standards must be meet to ensure a reduced rate of mortality in patients with AMI^3–8^.

The treatment of patients with AMI depends on the joint effort of the staff of the emergency department and those of the cardiac catheterization laboratory of hospitals. Emergency physicians and PCI surgeons must work together to complete the required intervention in a door-to-balloon (D2B) time of < 90 min, which substantially reduces the rate of mortality in patients with ST-elevation MI (STEMI). This requirement has been included in relevant guidelines for cardiology departments worldwide^9^. The importance of assessing the electrocardiographic readings of patients with STEMI has been indicated in the 2020 Advanced Cardiovascular Life Support guidelines^9^. These observations imply that emergency physicians have considerable influence on the survival rate of patients with STEMI. In the United States, the annual incidence of diagnostic errors among emergency department visits is more than 7 million, which accounts for 5.7% of all emergency department visits, and that of diagnostic errors leading to avoidable permanent disability or mortality in these patients is more than 350 000^10^. Therefore, reducing diagnostic errors in the emergency department is crucial for patient safety^10^. The correlation between the workload of emergency physicians and the rate of mortality in patients with AMI remains to be investigated.

A hospital revisit that occurs shortly after an emergency department visit (usually defined as a visit made within 72 h [3 days]) is referred to as an early return visit. The early return rate is regarded as a key indicator of the quality of emergency care^11–14^. According to US statistics pertaining to the period from 2010 to 2014, the rate of hospital readmission within 30 days after discharge among patients with STEMI was 12.3%^15^. Another large-scale, nationwide US study revealed that 10.3% of patients with STEMI who had multiple coronary arteries unblocked were hospitalized again within 30 days after discharge, and within this subgroup of patients with STEMI, 62.66% were hospitalized because of cardiac factors^16^. In the present study, we investigated whether the average daily volume of emergency physicians affects the risks of mortality within 30 days after a visit to the emergency department, revisit within 3 days after discharge from the hospital, and readmission within 14 days after discharge from the hospital in patients with STEMI undergoing PCI.

## Results

A total 27 219 patients with AMI were initially included. Of them, 231 patients were aged < 18 years, 3160 had been diagnosed as having STEMI, 89 died in accidents, 3091 were not hospitalized (patients with AMI [STEMI] should be hospitalized in Taiwan), and 2987 did not undergo PCI surgery. These patients were excluded. Thus, 17 661 patients with AMI were included in the final analysis.

We performed a bivariate analysis to investigate the primary outcomes. Most hospitals’ average volume of patients seen per day (PSPD) by emergency physicians fell into the Q1 to Q2 segment (27.30%; Table 1). Supplementary Table S1 shows the distribution of the average volume of PSPD by emergency physicians from 2012 to 2018. As shown in Table 2, non-significant differences between groups were noted in the rate of mortality within 30 days after visiting the emergency department (*P* = 0.716), the rate of revisit within 3 days (*P* = 0.293) and readmission within 14 days after discharge from the hospital (*P* = 0.376).

**Table 1:** Correlations between the average daily volume of emergency physicians and the characteristics of study population.

Variables	Total		Average daily volume of emergency physicians								
			≤ Q1		Q1 ~ Q2		Q2 ~ Q3		> Q3		*P* value
	N	%	N	%	N	%	N	%	N	%	
Total number	17,661	100.00	4,659	26.38	4,822	27.30	4,365	24.72	3,815	21.60	
PCI service model											< 0.001
Non-24-h	13,018	73.71	3,499	75.10	3,373	69.95	3,442	78.85	2,704	70.88	
24-h	4,643	26.29	1,160	24.90	1,449	30.05	923	21.15	1,111	29.12	
Gender											0.069
Female	2,899	16.41	780	16.74	825	17.11	719	16.47	575	15.07	
Male	14,762	83.59	3,879	83.26	3,997	82.89	3,646	83.53	3,240	84.93	
Age											< 0.001
< 45	1,885	10.67	486	10.43	483	10.02	492	11.27	424	11.11	
45–54	3,895	22.05	962	20.65	1,065	22.09	921	21.10	947	24.82	
55–64	4,958	28.07	1,240	26.62	1,352	28.04	1,317	30.17	1,049	27.50	
65–74	3,597	20.37	1,008	21.64	993	20.59	849	19.45	747	19.58	
75–84	2,431	13.76	680	14.60	685	14.21	581	13.31	485	12.71	
≥ 85	895	5.07	283	6.07	244	5.06	205	4.70	163	4.27	
Mean ± SD	61.88 ± 13.53		62.64 ± 13.93		62.13 ± 13.48		61.60 ± 13.51		60.98 ± 13.19		
CCI score^1^											0.004
0	6,914	39.15	1,754	37.65	1,855	38.47	1,710	39.18	1,595	41.81	
1	5,314	30.09	1,409	30.24	1,452	30.11	1,315	30.13	1,138	29.83	
2	2,069	11.72	548	11.76	577	11.97	500	11.45	444	11.64	
3	1,464	8.29	404	8.67	403	8.36	361	8.27	296	7.76	
≥ 4	1,900	10.76	544	11.68	535	11.09	479	10.97	342	8.96	
Monthly salary (NTD)											< 0.001
≤ 17,280	4,291	24.30	1,226	26.31	1,164	24.14	1,019	23.34	882	23.12	
17,281–22,080	2,625	14.86	671	14.40	732	15.18	677	15.51	545	14.29	
22,081–36,300	6,758	38.27	1,717	36.85	1,843	38.22	1,746	40.00	1,452	38.06	
≥ 36,301	3,987	22.58	1,045	22.43	1,083	22.46	923	21.15	936	24.53	
Urbanization level											< 0.001
Level 1	3,679	20.83	976	20.95	926	19.20	789	18.08	988	25.90	
Level 2	5,595	31.68	1,397	29.98	1,635	33.91	1,486	34.04	1,077	28.23	
Level 3	3,427	19.40	908	19.49	876	18.17	818	18.74	825	21.63	
Level 4	2,746	15.55	746	16.01	751	15.57	713	16.33	536	14.05	
Levels 5–7	2,214	12.54	632	13.57	634	13.15	559	12.81	389	10.20	
Other catastrophic illness											0.035
No	16,531	93.60	4,365	93.69	4,483	92.97	4,078	93.42	3,605	94.50	
Yes	1,130	6.40	294	6.31	339	7.03	287	6.58	210	5.50	
MI severity											0.152
1 blood vessel	15,475	87.62	4,041	86.74	4,230	87.72	3,833	87.81	3,371	88.36	
2 blood vessels	1,990	11.27	558	11.98	535	11.09	482	11.04	415	10.88	
≥ 3 blood vessels	196	1.11	60	1.29	57	1.18	50	1.15	29	0.76	
Coronary stent implantation											< 0.001
No	12,709	71.96	3,385	72.66	3,505	72.69	3,031	69.44	2,788	73.08	
Yes	4,952	28.04	1,274	27.34	1,317	27.31	1,334	30.56	1,027	26.92	
Triage level											< 0.001
Level 1	4,140	23.44	1,052	22.58	1,187	24.62	1,048	24.01	853	22.36	
Level 2	10,360	58.66	2,868	61.56	2,813	58.34	2,438	55.85	2,241	58.74	
Level 3	2,966	16.79	702	15.07	772	16.01	828	18.97	664	17.40	
Level 4 & 5	195	1.10	37	0.79	50	1.04	51	1.17	57	1.49	
Accumulated PCI operator volume											< 0.001
≤ Q1	4,577	25.92	1,415	30.37	1,302	27.00	1,097	25.13	763	20.00	
Q1 ~ Q2	4,364	24.71	1,085	23.29	1,298	26.92	1,037	23.76	944	24.74	
Q2 ~ Q3	4,396	24.89	1,035	22.22	1,193	24.74	1,096	25.11	1,072	28.10	
> Q3	4,324	24.48	1,124	24.13	1,029	21.34	1,135	26.00	1,036	27.16	
Previous year’s PCI operator volume											< 0.001
≤ Q1	4,728	26.77	1,509	32.39	1,376	28.54	1,115	25.54	728	19.08	
Q1 ~ Q2	4,408	24.96	1,114	23.91	1,307	27.10	1,062	24.33	925	24.25	
Q2 ~ Q3	4,146	23.48	966	20.73	1,200	24.89	1,033	23.67	947	24.82	
> Q3	4,379	24.79	1,070	22.97	939	19.47	1,155	26.46	1,215	31.85	
Hospital level											< 0.001
Medical centers	6,212	35.17	1,809	38.83	1,624	33.68	1,298	29.74	1,481	38.82	
Regional hospitals	9,666	54.73	2,118	45.46	2,723	56.47	2,729	62.52	2,096	54.94	
District hospitals	1,783	10.10	732	15.71	475	9.85	338	7.74	238	6.24	
Hospital ownership											< 0.001
Public	5,168	29.26	2,159	46.34	1,538	31.90	930	21.31	541	14.18	
Nonpublic	12,493	70.74	2,500	53.66	3,284	68.10	3,435	78.69	3,274	85.82	
Previous year’s PCI institutional volume											< 0.001
≤ Q1	4,721	26.73	1,407	30.20	1,273	26.40	1,287	29.48	754	19.76	
Q1 ~ Q2	4,306	24.38	1,148	24.64	1,264	26.21	1,002	22.96	892	23.38	
Q2 ~ Q3	4,328	24.51	1,260	27.04	1,118	23.19	981	22.47	969	25.40	
> Q3	4,306	24.38	844	18.12	1,167	24.20	1,095	25.09	1,200	31.45	

^1^Myocardial infarction was excluded from CCI score calculation.

**Table 2:** Correlations between average daily volume of emergency physicians and major outcomes of acute myocardial infarction.

Variables	Major outcomes								
	Death within 30 days			ED revisits within 3 days			Readmission within 14 days		
	Events	%	*P* value	Events	%	*P* value	Events	%	*P* value
Total number	1,131	6.40		130	0.74		178	1.01	
Average daily volume of emergency physicians			0.716			0.293			0.376
≤ Q1	299	6.42		26	0.56		42	0.90	
Q1 ~ Q2	295	6.12		38	0.79		43	0.89	
Q2 ~ Q3	293	6.71		39	0.89		53	1.21	
> Q3	244	6.40		27	0.71		40	1.05	
PCI service model			< 0.001			0.232			0.032
Non-24-h	883	6.78		102	0.78		144	1.11	
24-h	248	5.34		28	0.60		34	0.73	
Gender			< 0.001			0.268			0.442
Female	342	11.80		26	0.90		33	1.14	
Male	789	5.34		104	0.70		145	0.98	
Age (years)			< 0.001			< 0.001			0.034
< 45	45	2.39		7	0.37		13	0.69	
45–54	95	2.44		25	0.64		28	0.72	
55–64	188	3.79		32	0.65		49	0.99	
65–74	252	7.01		20	0.56		40	1.11	
75–84	338	13.90		27	1.11		34	1.40	
≧85	213	23.80		19	2.12		14	1.56	
Mean ± SD	72.30 ± 13.67			66.66 ± 14.80			65.52 ± 13.82		
CCI score^1^			< 0.001			0.019			0.004
0	241	3.49		34	0.49		48	0.69	
1	322	6.06		41	0.77		56	1.05	
2	185	8.94		22	1.06		24	1.16	
3	131	8.95		13	0.89		21	1.43	
≧4	252	13.26		20	1.05		29	1.53	
Monthly salary (NTD)			< 0.001			0.999			0.067
≦17,280	350	8.16		32	0.75		41	0.96	
17,281–22,080	192	7.31		19	0.72		26	0.99	
22,081–36,300	415	6.14		50	0.74		83	1.23	
≧36,301	174	4.36		29	0.73		28	0.70	
Urbanization level			< 0.001			0.002			0.004
Level 1	193	5.25		13	0.35		17	0.46	
Level 2	338	6.04		36	0.64		60	1.07	
Level 3	191	5.57		27	0.79		40	1.17	
Level 4	208	7.57		30	1.09		31	1.13	
Level 5–7	201	9.08		24	1.08		30	1.36	
Other catastrophic illness			< 0.001			0.092			0.084
No	976	5.90		117	0.71		161	0.97	
Yes	155	13.72		13	1.15		17	1.50	
MI severity			< 0.001			0.296			0.488
1 blood vessel	887	5.73		110	0.71		159	1.03	
2 blood vessels	220	11.06		20	0.91		19	0.87	
≥ 3 blood vessels	24	12.24		-	-		-	-	
Coronary stent implantation			< 0.001			0.499			0.234
No	748	5.89		97	0.76		121	0.95	
Yes	383	7.73		33	0.67		57	1.15	
Triage level			< 0.001			0.501			0.597
Level 1	489	11.81		33	0.80		45	1.09	
Level 2	469	4.53		72	0.69		106	1.02	
Level 3	161	5.43		25	0.84		27	0.85	
Levels 4 & 5	12	6.15		-	-		-	-	
Accumulated PCI operator volume			< 0.001			0.232			0.518
≤ Q1	471	10.29		43	0.94		52	1.14	
Q1 ~ Q2	236	5.41		25	0.57		41	0.94	
Q2 ~ Q3	203	4.62		32	0.73		48	1.09	
> Q3	221	5.11		30	0.69		37	0.86	
Previous year’s PCI operator volume			< 0.001			0.532			0.811
≤ Q1	463	9.79		41	0.87		46	0.97	
Q1 ~ Q2	225	5.10		31	0.70		49	1.11	
Q2 ~ Q3	223	5.38		25	0.60		43	1.04	
> Q3	220	5.02		33	0.75		40	0.91	
Hospital level			< 0.001			0.001			0.012
Medical centers	326	5.25		27	0.43		46	0.74	
Regional hospitals	683	7.07		83	0.86		106	1.10	
District hospitals	122	6.84		20	1.12		26	1.46	
Hospital ownership			0.587			0.705			0.609
Public	339	6.56		40	0.77		49	0.95	
Nonpublic	792	6.34		90	0.72		129	1.03	
Previous year’s PCI institutional volume			< 0.001			0.009			0.094
≤ Q1	344	7.29		39	0.83		59	1.25	
Q1 ~ Q2	309	7.18		44	1.02		47	1.09	
Q2 ~ Q3	254	5.87		29	0.67		40	0.92	
> Q3	224	5.20		18	0.42		32	0.74	

^1^Myocardial infarction was excluded from CCI score calculation.

Using a logistic regression model employing GEEs, we evaluated the effect of the average daily volume of emergency physicians on the risk of mortality within 30 days after visiting the emergency department in patients with AMI undergoing PCI. As shown in Table 3, with relevant variables controlled, the risk of mortality significantly increased (by 39%) in patients who received PCI for AMI when the average volume of PSPD or the average daily workload of the emergency physicians were > Q3 compared with the findings obtained when either of the aforementioned parameters were ≤ Q1 (odds ratio [OR]: 1.39; 95% confidence interval [CI]: 1.12 to 1.72; *P* = 0.003). The risk of mortality within 30 days after visiting the emergency department increased significantly, according to a risk trend analysis, with the increasing average volume of PSPD by emergency physicians (*P* = 0.032).

**Table 3:** Relative risks of major outcomes in patients with acute myocardial infarction undergoing percutaneous coronary intervention at different average daily volume of emergency physicians.

Variables	Death within 30 days			ED revisits within 3 days			Readmission within 14 days		
	aOR^1^	95% CI	*P* value	aOR^1^	95% CI	*P* value	aOR^1^	95% CI	*P* value
Average daily volume of emergency physicians (ref: ≤ Q1)									
Q1 ~ Q2	1.04	0.86–1.25	0.702	1.58	0.92–2.69	0.095	0.98	0.58–1.64	0.930
Q2 ~ Q3	1.22	0.98–1.51	0.077	1.85	1.18–2.89	0.007	1.34	0.83–2.16	0.229
> Q3	1.39	1.12–1.72	0.003	1.60	0.92–2.77	0.095	1.34	0.82–2.17	0.239
*P* for trend			0.032			0.297			0.241
24-h PCI model (ref: no)	0.94	0.75–1.20	0.638	1.01	0.65–1.56	0.969	0.84	0.53–1.32	0.453
Male (ref: female)	0.78	0.68–0.89	< 0.001	1.11	0.73–1.69	0.641	1.11	0.64–1.94	0.705
Age (years) (ref: < 45)									
45–54	0.96	0.66–1.39	0.826	1.64	0.71–3.83	0.250	1.02	0.50–2.09	0.948
55–64	1.42	1.06–1.89	0.018	1.59	0.68–3.69	0.286	1.32	0.62–2.83	0.468
65–74	2.33	1.67–3.25	< 0.001	1.29	0.52–3.20	0.586	1.37	0.64–2.93	0.415
75–84	4.44	3.23–6.11	< 0.001	2.36	0.95–5.83	0.064	1.63	0.76–3.52	0.211
≧85	8.41	5.73–12.32	< 0.001	4.67	1.88–11.60	< 0.001	1.84	0.95–3.59	0.072
CCI score^2^ (ref: 0)									
1	1.34	1.11–1.61	0.002	1.43	0.89–2.30	0.144	1.43	1.00–2.05	0.052
2	1.48	1.20–1.82	< 0.001	1.69	0.86–3.34	0.130	1.46	0.91–2.32	0.114
3	1.41	1.11–1.79	0.005	1.42	0.73–2.74	0.298	1.76	1.06–2.91	0.028
≥ 4	1.84	1.51–2.24	< 0.001	1.56	0.78–3.12	0.204	1.83	1.20–2.79	0.005
Monthly salary (ref: ≤ 17,280)									
17,281–22,080	0.86	0.71–1.04	0.114	0.93	0.49–1.76	0.821	0.94	0.56–1.60	0.832
22,081–36,300	0.78	0.66–0.92	0.004	0.98	0.66–1.45	0.912	1.27	0.87–1.85	0.221
≥ 36,301	0.76	0.64–0.91	0.002	1.29	0.77–2.16	0.333	0.87	0.51–1.47	0.595
Urbanization level (ref: Level 1)									
Level 2	1.12	0.91–1.39	0.275	1.68	0.85–3.32	0.134	1.94	1.17–3.22	0.011
Level 3	1.02	0.83–1.26	0.831	2.15	0.93–4.96	0.074	2.14	1.21–3.79	0.009
Level 4	1.11	0.90–1.37	0.316	2.54	1.10–5.88	0.030	1.76	0.92–3.40	0.090
Levels 5–7	1.26	0.98–1.62	0.067	2.62	1.08–6.40	0.034	2.04	1.08–3.83	0.027
Other catastrophic illness (ref: no)	1.59	1.25–2.02	< 0.001	1.35	0.72–2.53	0.351	1.21	0.72–2.01	0.476
MI severity (ref: 1 blood vessel)									
2 blood vessels	1.91	1.59–2.30	< 0.001	1.33	0.82–2.14	0.246	0.82	0.47–1.43	0.482
≥ 3 blood vessels	2.30	1.38–3.82	0.001	-	-	-	-	-	-
Coronary stent implantation (ref: no)	1.14	0.96–1.35	0.143	0.74	0.50–1.11	0.145	1.11	0.79–1.55	0.544
Triage level (ref: Level 1)									
Level 2	0.37	0.31–0.44	< 0.001	0.96	0.66–1.39	0.834	0.99	0.67–1.48	0.977
Level 3	0.37	0.30–0.47	< 0.001	0.96	0.57–1.60	0.96	0.71	0.42–1.22	0.212
Levels 4 & 5	0.39	0.20–0.78	0.008	-	-	-	-	-	-
Accumulated PCI operator volume (ref: ≤ Q1)									
Q1 ~ Q2	0.58	0.47–0.72	< 0.001	0.66	0.40–1.08	0.100	0.78	0.46–1.34	0.372
Q2 ~ Q3	0.54	0.41–0.70	< 0.001	0.87	0.51–1.46	0.592	0.89	0.52–1.53	0.677
> Q3	0.59	0.46–0.77	< 0.001	0.84	0.43–1.62	0.601	0.69	0.37–1.28	0.240
Previous year’s PCI operator volume (ref: ≤ Q1)									
Q1 ~ Q2	0.62	0.50–0.76	< 0.001	0.90	0.56–1.46	0.665	1.24	0.75–2.04	0.394
Q2 ~ Q3	0.68	0.56–0.81	< 0.001	0.74	0.48–1.16	0.186	1.13	0.65–1.99	0.665
> Q3	0.66	0.51–0.86	0.002	1.00	0.55–1.83	0.999	1.05	0.62–1.79	0.845
Hospital level (ref: medical centers)									
Regional hospitals	1.11	0.87–1.42	0.404	1.38	0.83–2.30	0.214	1.13	0.62–2.05	0.684
District hospitals	1.07	0.78–1.47	0.669	1.85	0.91–3.76	0.087	1.62	0.83–3.17	0.161
Hospital ownership (ref: public)									
Nonpublic	0.98	0.80–1.19	0.828	0.90	0.55–1.48	0.682	1.00	0.64–1.56	0.998
Previous year’s PCI institutional volume (ref: ≤ Q1)									
Q1 ~ Q2	1.11	0.92–1.35	0.275	1.32	0.84–2.09	0.230	0.92	0.58–1.44	0.701
Q2 ~ Q3	1.03	0.78–1.37	0.837	1.02	0.57–1.81	0.959	0.84	0.51–1.38	0.495
> Q3	0.94	0.72–1.24	0.668	0.67	0.32–1.43	0.304	0.69	0.34–1.41	0.310

^1^aOR, adjusted odds ratio.

^2^Myocardial infarction was excluded from CCI score calculation.

After performing the logistic regression, we investigated the effects of the average daily volume of emergency physicians on the risk of revisit within 3 days after discharge from the hospital in patients with AMI undergoing PCI. As shown in Table 3, with relevant variables controlled, the aforementioned risk significantly increased (by 85%) when the average volume of PSPD by emergency physicians was in the Q2 to Q3 segment compared with the findings obtained when this parameter was in the ≤ Q1 segment (OR 1.85; 95% CI 1.18 to 2.89; *P* = 0.007). To further confirm the accuracy of the primary outcome, the risk of mortality within 30 days after visiting the emergency department, we also conducted sensitivity analyses. From supplementary Table S2, it can be observed that regardless of using a binary, ternary, or quintile approach, the results align consistently with the findings of our study.

## Discussion

We found that the risk of mortality within 30 days after visiting the emergency department significantly increased when the workload of the emergency physicians was greater than the 75th percentile compared with the finding when this workload was at or less than the 25th percentile (*P* = 0.003). Thus, the risk of mortality increased with increasing workload (OR 1.04 to 1.39), probably because excessive workload prevented emergency physicians from taking care of all patients. In patients with STEMI with severe complications and likely atypical illness, precise diagnosis by emergency physicians is particularly crucial^17^. The effect of excessive workload on the risk of mortality may be attributed to the inability of emergency physicians to focus on all incoming patients^18–20^. Moreover, the overcrowding of emergency department prolongs the wait time for patients^21,22^ and thus may increase the risks of disability and mortality^23,24^. This is particularly true for patients with critical^19^ and acute illnesses (eg., acute coronary syndrome), which are heavily dependent on treatment timing^25^. Overcrowding also increases the D2B time^19^. Increases in both the ischemic time since symptom onset or the D2B time increase the risk of mortality in patients with STEMI^26,27^.

STEMI is diagnosed on the basis of patients’ clinical symptoms and electrocardiographic readings, and emergency physicians must have immediate and accurate interpretation of these readings. A study has reported a sensitivity value as low as 64.5% in emergency physicians’ evaluation of electrocardiographic readings of patients with STEMI^28^. Excessive workload compromises the immediacy and accuracy of evaluation, thus delaying PCI and increasing mortality risk^27,29,30^. Diagnostic errors in the emergency department are a major concern, particularly for patients with atypical symptoms and when the emergency department is overcrowded^10^. Effective policies should be designed and implemented to reduce these errors. Disease prognosis can be significantly improved by providing early assessment and intervention after symptom onset. Efforts in this regard should include the coordination between hospitals and the prearrival medical care system^31^. Because of excessive workload, emergency physicians are often unable to provide prearrival instructions, which negatively affects disease prognosis.

We found that the risk of revisit within 3 days after discharge from the hospital significantly increased in patients who received PCI for AMI when the average daily workload of emergency physicians was in the Q2 to Q3 segment compared with the findings obtained when this parameter was in the ≤ Q1 segment (OR 1.85). A Taiwanese study reported that disease-related factors are the primary reasons (in 80.9% of all cases) for revisiting the emergency department within 3 days after discharge; this is followed by patient-related factors (10.9%) and then physician-related factors (8.2%). Misdiagnosis accounts for 3.7% of all physician-related reasons for revisiting the emergency department after discharge^32^. Excessive workload reduces the ability of emergency physicians to attend to patients due to the constant distractions by incoming patients^18–20^, which possibly increases the rate of medical errors and the risk of revisits within 3 days after discharge from the hospital. Moreover, overcrowding leads to impatience and thus patients often leave the department before receiving appropriate treatment, although some of these patients might need inpatient treatment^33–35^. This may increase the risk of revisit within 3 days after discharge from the hospital. Overcrowding may also delay treatment and cause additional medical errors^24^, which, in turn, increases the aforementioned risk.

Patients with AMI who had a history of diabetes, hypertension, or heart failure; a family history of coronary artery disease; or a lower socioeconomic status have significantly higher risks of readmission after discharge from the emergency department than those who did not^15^. Because of data limitations, we could not investigate the family history and health behaviors of our cohort. Future studies are warranted to incorporate relevant factors in the analysis.

The present study has several strengths. Taiwan’s National Health Insurance program provides insurance coverage to approximately 99.9% of its citizens. Therefore, the data from the National Health Insurance Research Database provided a sufficient representation of Taiwan’s population. National, full-population, and multiyear data were applied in the present study; hence, the statistics were reliable. Furthermore, we comprehensively explored the factors influencing the prognosis of patients with AMI (eg., the primary outcomes of this study). To the best of our knowledge, this study is first to investigate the possible effects of the workload of emergency physicians on the prognosis of AMI in a cohort with relevant variables controlled. We also conducted sensitivity analyses carefully to reconfirm the accuracy of the primary outcome, the risk of mortality within 30 days after visiting the emergency department, aiming to enhance the validity and reliability of the study. The results revealed that regardless of the classification method, the group with the highest average volume of PSPD by emergency physicians consistently exhibited a significantly higher risk of mortality within 30 days after visiting the emergency department compared to the group with the lowest average volume of PSPD by emergency physicians. This finding may also underscore the importance of an appropriate workload for emergency physicians.

The present study has some limitations. We performed a retrospective analysis by using secondary data obtained from the National Health Insurance Research Database. Because of the data constraints of the database, we could not study the health behaviors of the patients, thrombolysis in myocardial infarction (TIMI) score and D2B data. In addition, most emergency rooms are open 24/7 in Taiwan and the actual number of work hours per day for emergency physicians ranges from 8 to 12. Therefore, the shift change of a part of the emergency physician may involve transitions across days, such as on the graveyard shift. The PSPD calculation of emergency physicians may be biased in the present study, due to the shift work. Last, since this study focuses on discussing STEMI patients undergoing cardiac catheterization, patients who died upon arrival at the emergency department or before undergoing cardiac catheterization were excluded. This could potentially lead to bias due to the exclusions.

In the clinical setting, we have always placed significant emphasis on the prognosis of STEMI patients. Especially when we evaluate and promptly perform emergency cardiac catheterization for STEMI patients in the emergency department, the prognosis of each STEMI patient may not always meet our expectations. In addition to the conclusions mentioned in previous research, understanding and suggesting ways to improve the prognosis of STEMI patients have always been our concern from a public health and policy perspective. In the current emergency capacity grading system in Taiwan, it is stipulated that for every 5,000 emergency department visits annually, one full-time emergency physician should be added. Yet, we do not have clear evidence to demonstrate its rationale. Therefore, despite numerous research limitations, we still aim to determine a reasonable range for the appropriate workload of emergency physicians, serving as a reference for policy-making.

## Conclusions

Patients with acute or severe diseases, such as AMI, must be treated with focus and accurate judgment. Excessive workload of attending emergency physicians may lead to grave errors. Thus, their workload must be regulated to reduce the risks of mortality, revisit, and readmission after discharge from the hospital in these patients. Our study may serve as a reference for making relevant policies.

## Material and methods

### Data sources

The data analyzed in the present study were secondary data. Data for the period from 2010 to 2018 were retrieved from the National Health Insurance Research Database for analysis. We collected data files including outpatients, emergency visits, inpatients, catastrophic illnesses, and death cause statistics. These data were compiled, and those that corresponded to the relevant variables were selected for analysis. Since the patient identifications in the National Health Insurance Research Database have been scrambled random identification numbers for insured patients by the Taiwan government for academic research use, the informed consent was waived by the Research Ethics Committee of the Taichung Jen-Ai Hospital. The research was conducted in accordance with the 1964 Declaration of Helsinki and amendments and was approved by the institutional review board (IRB) of the Taichung Jen-Ai Hospital (IRB NO. 111–20), Taiwan.

### Study participants

**Figure 1:**
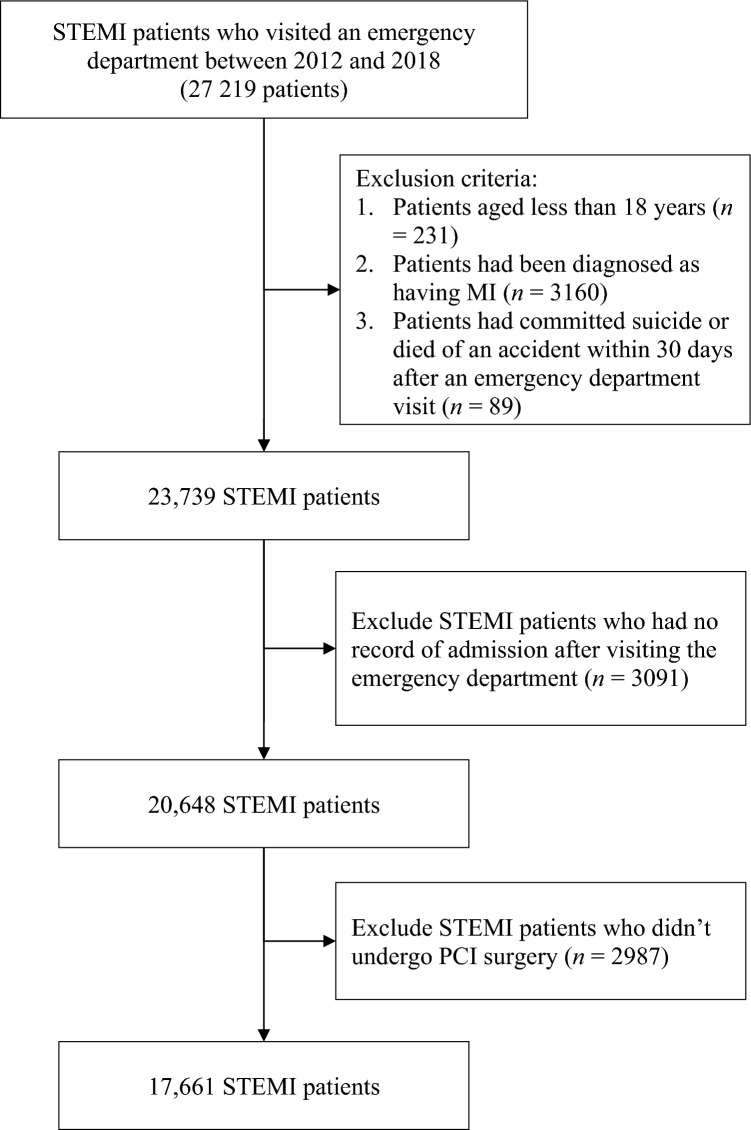
Flowchart for patient selection.

This study included patients with AMI who visited emergency departments between 2012 and 2018. Patients were excluded if they were aged < 18 years, had been diagnosed as having MI, had committed suicide or died in an accident within 30 days after visiting the emergency department, had no record of admission after visiting the emergency department, or had not undergone PCI. The emergency department visits were defined as medical behaviors that corresponded to the billing codes 00201B, 00202B, 00203B, 00204B, and 00225B. A diagnosis of AMI was defined as a diagnosis made with the following codes: 410.0X–410.6X and 410.8X of ICD-9-CM or I21.01, I21.02, I21.09, I21.11, I21.19, I21.21, I21.29, I22.0, I22.1, I22.8, and I22.9 of ICD-10-CM. Figure 1 depicts the flowchart for patient selection.

### Definition and description of variables

The definition of the variables considered in the present study are as follows. We defined 24-h PCI service as a treatment performed in a hospital offering PCI service with 24 h per day. In Taiwan, the evaluation of emergency capacity grading is conducted by the Joint Commission of Taiwan. According to the emergency medical capabilities of each hospital, they are categorized into three levels. In this study, hospitals providing 24-h PCI service were defined as Level I hospitals according to the Emergency Capability Grading. In Taiwan's hospital accreditation system, the guidelines state that ‘Level I hospitals are required to provide 24-h cardiac catheterization services and achieve a D2B time of less than 90 min for emergency treatment of STEMI patients in over 75% of cases.’ A patient with STEMI was defined as a patient who was diagnosed with STEMI in accordance with the following the primary discharge diagnosis codes: 410.0X–410.6X and 410.8X of ICD-9-CM or I21.01, I21.02, I21.09, I21.11, I21.19, I21.21, I21.29, I22.0, I22.1, I22.8, and I22.9 of ICD-10-CM. The sex of the patients was either male or female. The patients were divided into the following age groups: < 45, 45 to 54, 55 to 64, 65 to 74 years, 75 to 84 years, and ≥ 85 years. For monthly salary, the patients were divided into the following groups: ≤ NT$17 280, NT$17 281 to NT$22 080, NT$22 081 to NT$36 300, and ≥ NT$36 301. The urbanization levels of the patients’ insured areas were divided into Levels 1 to 7; Levels 1 and 7 indicated the highest and lowest levels of urbanization, respectively^36^.

The hospitals investigated in the present study were classified as medical centers regional hospitals, and district hospitals. On the basis of ownership, the hospitals were classified as public and nonpublic hospitals. The comorbidity severity was estimated using Deyo’s Charlson comorbidity index (CCI). Deyo CCI is a modified version of the CCI, and it classifies comorbidities into 17 types. Patients’ primary and secondary diagnostic codes within 1 year before their visit to the emergency department were converted into weighted scores and then summed to calculate the corresponding Deyo CCI scores^37^. The comorbidity severity was rated on a scale from 0 to 4 points. Classification of catastrophic illnesses comprised the presence (yes) and absence (no) of any major illness. The applied triage categories were Level 1 (Code 00201B), Level 2 (Code 00202B), Level 3 (Code 00203B), Level 4 (Code 00204B), and Level 5 (Code 00225B); Level 1 patients were those who most urgently required medical attention. Administration of PCI referred to patients who underwent PCI (codes 33076B, 33077B, and 33078B) during emergency visits or hospitalization.

The annual volume of PCI used for each hospital was within one of the quartile ranges (≤ Q1, Q1 to Q2, Q2 to Q3, and > Q3) established based on the PCI reimbursement claims by hospitals across the country. MI severity was evaluated on the basis of the number of blood vessels operated during PCI; considering this, patients were stratified into the following 3 groups: 1 blood vessel (code, 33076B), 2 blood vessels (code, 33077B), and ≥ 3 blood vessels (code, 33078B). On the basis of stent use, patients were stratified into the with-stent and without-stent groups. In the relevant studies, the volume of PCI was divided into high, medium, and low levels^5,7^. In another study, the quartile method was used to precisely analyze the effects of the workload of admitting physicians^8^. The average volume of patients seen per day (PSPD) by emergency physicians was calculated by dividing the volume of reimbursement claims for emergency treatment on the day of patient admission by the number of emergency physicians on that day. Each hospital’s PSPD by emergency physicians fell into one of the quartile ranges (≤ Q1, Q1 to Q2, Q2 to Q3, and > Q3) established based on the average volume of PSPD by emergency physicians across the country on the corresponding day. The cumulative volume of reimbursement claims for PCI performed by each PCI surgeon was evaluated for the period between 2010 and the day of undergoing PCI. The volume fell into one of the quartile ranges (≤ Q1, Q1 to Q2, Q2 to Q3, and > Q3) established based on the cumulative volume of reimbursement claims for PCI performed by all PCI surgeons in Taiwan in the corresponding year. The volume of reimbursement claims for PCI performed by each PCI surgeon in the preceding year was evaluated using data corresponding to the 1-year period preceding the day of undergoing PCI. The volume would fall into one of the quartile ranges (≤ Q1, Q1 to Q2, Q2 to Q3, and > Q3) established based on the preceding year’s annual volume of PCIs performed by all PCI surgeons across the country. The annual hospital volume of PCIs performed in each hospital in the preceding year was evaluated using data corresponding to the 1-year period preceding the day of PCI and fell into one of the quartile ranges (≤ Q1, Q1 to Q2, Q2 to Q3, and > Q3) established based on the preceding year’s annual volume of PCIs performed in all hospitals across the country. In this study, important relevant variables were selected based on previous research, and potential influencing factors were included in the model to control for their effects. In terms of the patients' conditions, we incorporated age, gender, CCI score, and other catastrophic illnesses. For socioeconomic status, we controlled for monthly salary and urbanization level. Regarding disease severity, we controlled for factors such as the number of blocked coronary vessels, stent placement, and emergency triage level. At the hospital characteristics, we controlled for whether 24-h cardiac catheterization services were available, hospital level, hospital ownership, and the previous year’s PCI institutional volume. Additionally, for the workload and experience of the cardiac catheterization operators, we controlled for accumulated PCI operator volume and the previous year’s PCI operator volume. After controlling for the above significant variables in the study, we discussed the association between emergency physicians’ workload and AMI outcomes.

### Assessment of major outcomes

We primarily explored whether the average daily volume of emergency physicians affected the risks of mortality within 30 days after visiting the emergency department, revisit within 3 days after discharge from the hospital, and readmission within 14 days after discharge from the hospital in patients with AMI undergoing PCI.

### Statistical analysis

The present study adopted a retrospective cohort design. SAS 9.4 (SAS Institute, Cary, NC, USA) was employed for secondary data processing and statistical analysis. The following descriptive data are presented in terms of number and percentage values: age, sex, monthly salary, urbanization level, CCI score, catastrophic illnesses, triage level, coronary stent implantation, MI severity, mortality within 30 days after visiting the emergency department visit, revisit within 3 days after discharge from the hospital, readmission within 14 days after discharge from the hospital, hospitals’ ability to offer 24-h PCI service, hospital level, hospital ownership.

Inferential statistics were used to investigate the primary outcomes. First, a chi-square test was performed to analyze the differences in the primary outcomes between subgroups with different average daily volumes of emergency physicians. Subsequently, a logistic regression model employing generalized estimating equations (GEEs) was used to compare the subgroups in terms of the primary outcomes after adjusting for covariates (eg. age, sex, monthly salary, urbanization level, CCI score, catastrophic illnesses, hospital level, hospital ownership, and triage level). The logistic regression with GEE model was also used to reduce hospital cluster effects.

## Data Availability Statement

The National Health Insurance Database used to support the findings of this study were provided by the Health and Welfare Data Science Center, Ministry of Health and Welfare (HWDC, MOHW) under license and so cannot be made freely available. Requests for access to these data should be made to HWDC (https://dep.mohw.gov.tw/dos/np-2497-113.html).

### Supplementary Information

Supplementary Information.
