# Supplementary material for: The association between the workload of emergency physicians and the outcomes of acute myocardial infarction: a population-based study
Source: Sci Rep. 2023 Dec 1;13:21212. doi: 10.1038/s41598-023-48150-0 (PMC10692142; doi:10.1038/s41598-023-48150-0)
Supplement: Supplementary file 1 — Supplementary Information. [file 41598_2023_48150_MOESM1_ESM.docx]

**Supplementary Materials:**

**Supplementary Table S1.** The distribution of the average volume of patients seen per day by emergency physicians.

| **Year** | **Mean** | **Standard**  **Deviation** | **Minimum** | **Maximum** | **Percentile** | | | | | | | | |
| --- | --- | --- | --- | --- | --- | --- | --- | --- | --- | --- | --- | --- | --- |
|  |  |  |  |  | **20^th^** | **25^th^** | **33^th^** | **40^th^** | **50^th^** | **60^th^** | **66^th^** | **75^th^** | **80^th^** |
| 2012 | 17 | 7 | 3 | 67 | 12 | 12 | 13 | 14 | 15 | 17 | 19 | 21 | 22 |
| 2013 | 17 | 7 | 3 | 60 | 12 | 12 | 13 | 14 | 16 | 17 | 19 | 22 | 23 |
| 2014 | 17 | 7 | 5 | 53 | 11 | 12 | 13 | 14 | 15 | 17 | 19 | 21 | 23 |
| 2015 | 17 | 7 | 6 | 85 | 12 | 12 | 13 | 14 | 16 | 17 | 19 | 21 | 23 |
| 2016 | 18 | 8 | 6 | 65 | 11 | 12 | 13 | 15 | 16 | 18 | 19 | 22 | 24 |
| 2017 | 18 | 8 | 5 | 73 | 11 | 12 | 13 | 14 | 16 | 17 | 19 | 21 | 22 |
| 2018 | 17 | 8 | 4 | 65 | 11 | 12 | 13 | 14 | 15 | 17 | 18 | 21 | 22 |
| 2012~2018 | 17 | 8 | 3 | 85 | 11 | 12 | 13 | 14 | 16 | 17 | 19 | 21 | 23 |

**Supplementary Table S2.** Sensitivity analysis of the risk of mortality within 30 days at different average daily volumes of emergency physicians.

| **Average daily volume**  **of emergency physicians** | **Death within 30 days** | | | | | | |
| --- | --- | --- | --- | --- | --- | --- | --- |
|  | **Events** | **%** | **aOR ^1^** | **95% CI** | | | ***P* value** |
| An interval into two equal parts |  |  |  |  |  |  |  |
| ≤Q2 (ref.) | 594 | 6.27 | 1 |  |  |  |  |
| >Q2 | 537 | 6.56 | 1.26 | 1.09 | - | 1.46 | 0.002 |
| *P* for trend |  |  |  |  |  |  | 0.417 |
| An interval into three equal parts |  |  |  |  |  |  |  |
| ≤ 33^th^ percentile (ref.) | 380 | 6.39 | 1 |  |  |  |  |
| 33^th^－66^th^ percentile | 388 | 6.41 | 1.10 | 0.93 | - | 1.29 | 0.265 |
| > 66^th^ percentile | 363 | 6.41 | 1.27 | 1.06 | - | 1.52 | 0.010 |
| *P* for trend |  |  |  |  |  |  | 0.966 |
| An interval into five equal parts |  |  |  |  |  |  |  |
| ≤ 20^th^ percentile (ref.) | 215 | 6.19 | 1 |  |  |  |  |
| 20^th^－40^th^ percentile | 246 | 6.56 | 1.14 | 0.94 | - | 1.39 | 0.175 |
| 40^th^－60^th^ percentile | 189 | 5.93 | 1.12 | 0.89 | - | 1.40 | 0.336 |
| 60^th^－80^th^ percentile | 272 | 6.94 | 1.34 | 1.07 | - | 1.69 | 0.011 |
| >80^th^ percentile | 209 | 6.28 | 1.46 | 1.14 | - | 1.86 | 0.003 |
| *P* for trend |  |  |  |  |  |  | 0.630 |

^1^ Abbreviations: aOR, adjusted odds ratio. Extraneous factors adjusted in the model were PCI service model, gender, age, CCI score, monthly salary, the urbanization of insured areas, other catastrophic illness, MI severity, coronary stent implantation, accumulated PCI operator volume, previous year’s PCI operator volume, hospital level, hospital ownership, and previous year’s PCI institutional volume.
